# Taxonomic assessment of species of the genus *Octopus* from the northeastern Pacific via morphological, molecular and morphometric analyses

**DOI:** 10.7717/peerj.8118

**Published:** 2019-11-29

**Authors:** Mariana Díaz-Santana-Iturrios, César Augusto Salinas-Zavala, Francisco Javier García-Rodríguez, Jasmín Granados-Amores

**Affiliations:** 1 Programa de Doctorado en Ciencias Biológico Agropecuarias, Universidad Autónoma de Nayarit, Xalisco, Nayarit, Mexico; 2 Centro de Investigaciones Biológicas del Noroeste (CIBNOR), La Paz, Baja California Sur, Mexico; 3 Instituto Politécnico Nacional, Centro Interdisciplinario de Ciencias Marinas (IPN-CICIMAR), La Paz, Baja California Sur, Mexico; 4 Escuela Nacional de Ingeniería Pesquera, Universidad Autónoma de Nayarit, San Blas, Nayarit, Mexico

**Keywords:** Octopod, Taxonomy, Species discrimination, Octopodidae, Taxonomic problems, Octopuses, Taxonomic tools

## Abstract

Species of the genus *Octopus* from the northeastern Pacific are ecologically and economically important; however, their taxonomy is confusing and has not been comprehensively assessed. In this study, we performed a taxonomic evaluation of these species considering the morphological characteristics of the original descriptions, a molecular analysis of partial COI-gene sequences, and a traditional morphometry analysis of nine body measurements. Several interesting findings were obtained with our results: for instance, we updated the diagnoses of some species by including characters such as the number of lamellae per demibranch and the presence of chromatophores in the visceral sac; we deposited partial COI-gene sequences of species that had not been incorporated into the GenBank repository; and according to the morphometric analysis, we confirmed that the lengths of arms I–IV are relevant to discriminate the species under study. The taxa evaluated were morphologically, molecularly and morphometrically well-delimited; however, features such as funnel organ shape and arm length proportions in regard to dorsal mantle length are either not included in the diagnosis of the genus *Octopus* or overlap with other genera. Hence, this information, combined with the results obtained from the molecular analysis, supports the generic re-assignation of two of the species evaluated.

## Introduction

Octopuses are important marine resources worldwide; however, their taxonomy is complicated (Norman & Hochberg, 2005; FAO, 2016). Hence, relevant studies have been developed in attempts to solve the taxonomic complexity of different groups of the order Octopoda Leach, 1818 (Taki, 1964; Norman & Hochberg, 2005; Allcock et al., 2011; De Luna Sales et al., 2019). Nevertheless, there is a considerable number of taxa that remain unrevised or little information was added after their description (Voss et al., 1998; Amor et al., 2016), such is the case for octopodids of the genus *Octopus* Cuvier, 1797 (Jereb et al., 2016).

Similar to the rest of cephalopods and most animal species, the taxonomy of the genus *Octopus* is fixed on the basis of morphological and meristic attributes of body parts. For species of this genus, the relevant characters include: number of gill lamella, funnel organ shape, presence or absence of ocelli, among others (Verrill, 1883; Pickford & McConnaughey, 1949; Berry, 1953). However, in many species (e.g., confusion between *Octopus insularis* Leite & Haimovici, 2008 and *Octopus vulgaris* Cuvier, 1797, see Lima et al. (2017)), the morphological traits are not well-delineated and this hinders their identification. To increase this problematic, any species with an ink sac and arms in a two-row sucker arrangement is catalogued within the genus, and despite that these two characters are considered diagnostic for *Octopus*, these attributes are also detected in other genera of the family Octopodidae (Cuvier, 1797; Guzik, Norman & Crozier, 2005; Strugnell et al., 2014; Jereb et al., 2016). In addition, the milestone of the taxonomic complexity of the genus *Octopus* is the type species of this taxon, *Octopus vulgaris*, which comprises a complex of morphologically similar but genetically distinct species catalogued in types (Amor et al., 2019).

In the northeastern Pacific, the lack of consistent identification is detected in research papers (e.g., identification of “*Octopus macropus* (Risso, 1826)” in Alejo-Plata et al., 2014) and fishery statistics (https://www.gob.mx/sader), which can limit the conservation and sustainable use of octopuses as living organisms and as fishery resources. Despite the fact that relevant research regarding some of these taxa was developed in recent years (Domínguez-Contreras et al., 2018), information is still scarce, especially from taxonomic studies. Thus, it is important to revise the morphological characteristics of octopuses from the northeastern Pacific and include attributes from quantitative sources.

Eleven species of the genus *Octopus* can be found in this area, *Octopus bimaculatus* Verrill, 1883, *Octopus bimaculoides* Pickford & McConnaughey, 1949 and *Octopus hubbsorum* Berry, 1953 are commercially important, *Octopus chierchiae* Jatta, 1889, *Octopus fitchi* Berry, 1953, *Octopus micropyrsus* Berry, 1953 and *Octopus penicillifer* Berry, 1954 are considered rare, and *Octopus californicus* Berry, 1911, *Octopus alecto* Berry, 1953, *Octopus rubescens* Berry, 1953 and *Octopus veligero* Berry, 1953 are provisionally assigned to the genus (Hochberg, 1998; Norman & Hochberg, 2005; Jereb et al., 2016). With the exception of *O. rubescens* and *O. californicus* (diagnoses detailed by Hochberg, 1998), the taxonomy of these taxa has not been updated after their description. Thus, in order to corroborate the number of taxa in the northeastern Pacific and mitigate the taxonomic problems mentioned earlier, in this research we performed a taxonomic revision of species of the genus *Octopus* via morphological comparisons, COI-sequence analysis and traditional morphometrics.

## Materials and Methods

Over a period of 12 years, a total of 270 individuals were collected in oceanographic cruises and artisanal fisheries in the coast of the Mexican Pacific, including the Gulf of California, in compliance with the regulations stated in NOM-059-SEMARNAT-2010 (Table 1). Octopuses were identified to the species level using the morphological characteristics of the following original descriptions: Verrill (1883), Jatta (1889), Berry (1911), Pickford & McConnaughey (1949), Berry (1953) and Berry (1954) and the catalog by Jereb et al. (2016).

**Table 1:** Sampling data of octopuses of the genus *Octopus* from the northeastern Pacific and sample size for each analysis.

Species	Sampling date	Location-State	DML range (cm)	Preservation method	Sampling method	No. of individuals
*Octopus bimaculatus*	Feb-21-2016	San Juanico-Baja California Sur	9.5–15.5	Frozen	Hook/Fishery	30
*Octopus chierchiae*	2006	Gulf of California-Baja California Sur	6.7	*Formaldehyde 4%/Ethanol 96°	Net/Cruise	1
	2006	Bahia Magdalena-Baja California Sur	7	*Formaldehyde 4%/Ethanol 96°	Net/Cruise	1
*Octopus californicus*	2004	Bahia Magdalena-Baja California Sur	2.5, 3.9	*Formaldehyde 4%/Ethanol 96°	Net/Cruise	2
	2005	Bahia Magdalena-Baja California Sur	4.9–6.1	*Formaldehyde 4%/Ethanol 96°	Net/Cruise	3
	2011	Bahia Magdalena-Baja California Sur	8–13.6	*Formaldehyde 4%/Ethanol 96°	Net/Cruise	31
*Octopus bimaculoides*	Sep-2016	Guerrero Negro-Baja California Sur	7–14	Frozen	Hook/Fishery	50
*Octopus hubbsorum*	Feb-04-2014	Bahia de Matanchen-Nayarit	7–8	Frozen	Hook/Fishery	3
	Sep-07-2014	San Bruno-Baja California Sur	14.1–15	Frozen	Hook/Fishery	4
	Nov-2014	Acapulco-Guerrero	7.1–10	Frozen	Hook/Fishery	3
	Jan-05-2015	Mazatlan-Sinaloa	8.8–11.8	Frozen	Hook/Fishery	4
	Feb-03-2015	Bahia Magdalena-Baja California Sur	10–18.5	Frozen	Hook/Fishery	21
	Feb-21-2016	San Juanico-Baja California Sur	13.2–15.3	Frozen	Hook/Fishery	4
	Mar-2016	Melaque-Jalisco	8–11	Frozen	Hook/Fishery	4
	Mar-2016	Santa Rosalia-Baja California Sur	9.5–14.1	Frozen	Hook/Fishery	3
	May-2016	Guaymas-Sonora	6.1–12.3	Frozen	Hook/Fishery	4
*Octopus alecto*	Aug-08-2015	Tobari-Sonora	2–7.5	Frozen	Hook/Fishery	50
*Octopus veligero*	2004	Bahia Magdalena-Baja California Sur	3.7–5.4	Frozen	Net/Cruise	8
	2005	Bahia Magdalena-Baja California Sur	3.7–8.4	Frozen	Net/Cruise	13
	2007	Bahia Magdalena-Baja California Sur	2.7–4.4	Frozen	Net/Cruise	2
	2011	Bahia Magdalena-Baja California Sur	7–12.7	Frozen	Net/Cruise	7
	2012	Bahia Magdalena-Baja California Sur	3.2–11.3	Frozen	Net/Cruise	12
	2015	Bahia Magdalena-Baja California Sur	3.5–9.2	Frozen	Net/Cruise	8
*Octopus micropyrsus*	2012	Guerrero Negro-Baja California Sur	6.3–6.7	*Formaldehyde 4%/Ethanol 96°	Hook/Fishery	2
**N Total**						**270**
Morphological analysis						270
COI sequence analysis						29
Morphometric analysis						266

**Note:**

* It is possible that the samples were fixed at some point in formaldehyde 4% due to their smell, although tissue was not fully impregnated by this substance.

A small piece of tissue was removed from the arm (the central portion of each transversal piece) of 29 individuals that represented the species identified morphologically, these samples were rinsed with distilled water and 96% ethanol and placed in vials filled with 96% ethanol. DNA was purified and extracted using QIAGEN^®^ DNeasy Blood & Tissue kit, following the steps of the protocol for animal tissue (spin-column). A fragment of COI was amplified using the primers developed by Folmer et al. (1994) (LCO1490 and HCO2198). Amplifications were conducted at 25 µl reactions consisting of 2.5 µl of Buffer *Taq* (10X -mg) invitrogen™, 0.5 µl of dNTPs (10 mM) invitrogen™, 1.2 µl of each primer (10 µM), 16.35 µl of Milli-Q H_2_O, 2 µl of MgCl_2_ (50 mM) invitrogen™, 0.25 µl of *Taq* polymerase (5 U/µl) invitrogen™ and 1 µl of extracted DNA. The thermal cycler conditions were the following: 3 min at 96 °C for denaturation, followed by 40 cycles of 30 s at 95 °C, 45 s at 50 °C and 1 min at 72 °C, and a final extension of 5 min at 72 °C. All amplified products were sequenced in both directions with the same primers used for PCR (MACROGEN Inc., Seoul, South Korea). The sequences obtained were assembled and edited using BioEdit 7.2.6 software (Hall, 1999). Edited sequences were deposited in GenBank (Accession numbers: MK649783–MK649811). Additional DNA sequences (COI fragments) were obtained from GenBank and included in the analyses for comparative purposes (Table 2). These sequences belonged to one species (preferably the type) of each genus of the family Octopodidade, except for *Bathypurpurata* Vecchione, Allcock & Piatkowski 2005, *Euaxoctopus* Voss, 1971, *Galeoctopus* Norman, Boucher & Hochberg 2004, *Histoctopus* Norman, Boucher-Rodoni & Hochberg, 2009, *Macrochlaena* Robson, 1929, *Microeledone* Norman, Hochberg & Boucher-Rodoni, 2004, *Pteroctopus* Fischer, 1882, *Sasakiopus* Jorgensen, 2009, *Teretoctopus* Robson, 1929, *Tetracheledone* Voss, 1955 and *Vosseledone* Palacio, 1978, which were not represented in GenBank. In addition, a partial COI gene sequence of *Opisthoteuthis depressa* Ijima & Ikeda, 1895 was used as outgroup (Table 2). Sequences were aligned by the ClustalW algorithm (Thompson, Higgins & Gibson, 1994) in BioEdit 7.2.6 software. The phylogenetic relationships among octopuses were reconstructed in order to represent the species identification based on COI sequences using a Bayesian Inference analysis in Mr. Bayes v3 (Huelsenbeck & Ronquist, 2001) with the GTR+G model (Tavaré, 1986), selected by BIC in Mega 7 software (Kumar, Stecher & Tamura, 2016). The Bayesian analysis was performed with four default heated chains, running 1,000,000 generations of the Markov Chain Monte Carlo (MCMC) and saving at every 1,000th generation. The first 1,000 trees were discarded as burn-in and the consensus tree was visualized and edited in FigTree 1.4.4 software. In addition, Bayesian Poisson Tree Processes (bPTP) (Zhang et al., 2013) were employed to infer molecular clades based on the inferred molecular phylogeny. This analysis (bPTP) was conducted on the species delimitation web server https://species.h-its.org/.

**Table 2:** Partial COI-gene sequences of octopuses obtained from GenBank and used in this study.

Species	Accession numbers
*Abdopus aculeatus* (d’Orbigny, 1834)	GQ900726.1
*Adelieledone polymorpha* (Robson, 1930)	GU073668.1
*Ameloctopus litoralis* (Norman, 1992)	HM104255.1
*Amphioctopus membranaceus* (Quoy & Gaimard, 1832)	MH293068.1
*Bathypolypus arcticus* (Prosch, 1847)	AF000029.1
*Bentheledone* sp. (Robson, 1932)	AF377975.1
*Benthoctopus thielei* (Robson, 1932)	HM572185.1
*Callistoctopus ornatus* (Gould, 1852)	MK593419.1
*Cistopus indicus* (Rapp, 1835)	KC409359.1
*Eledone moschata* (Lamarck, 1798)	MH293105.1
*Enteroctopus megalocyathus* (Gould, 1852)	HM572175.1
*Graneledone verrucosa* (Verrill, 1881)	AF000042.1
*Grimpella thaumastocheir* (Robson, 1928)	HM104259.1
*Hapalochlaena lunulata* (Quoy & Gaimard, 1832)	AB191278.1
*Macrotritopus* sp. (Grimpe, 1922)	MG778072.1
*Megaleledone setebos* (Robson, 1932)	GU073581.1
*Muusoctopus longibrachus* (Ibáñez, Sepúlveda & Chong, 2006)	KM459478.1
*Pareledone charcoti* (Joubin, 1905)	AF377971.1
*Paroctopus digueti* (Perrier & Rochebrune, 1894)	KT335833.1
*Praealtus paralbida* (Allcock, Collins, Piatkowski & Vecchione, 2004)	HM104261.1
*Robsonella fontaniana* (d’Orbigny, 1834)	KF774313.1
*Scaeurgus unicirrhus* (Delle-Chiaje, 1839–1841)	HM104263.1
*Thaumeledone peninsulae* (Allcock, Collins, Piatkowski & Vecchione, 2004)	EU071446.1
*Thaumoctopus mimicus* (Norman & Hochberg, 2005)	MK410934.1
*Velodona togata* (Chun, 1915)	EU071447.1
*Vulcanoctopus hydrothermalis* (González & Guerra, 1998)	HM572181.1
*Wunderpus photogenicus* (Hochberg, Norman & Finn, 2006)	GQ900748.1
*Opisthoteuthis depressa* (Ijima & Ikeda, 1895)	AB191282.1
*Octopus alecto* * Berry, 1953	MK649783–MK649786
*Octopus bimaculatus* * Verrill, 1883	MK649787–MK649791
*Octopus californicus* * Berry, 1911	MK649792–MK649795
*Octopus hubbsorum* * Berry, 1953	MK649796–MK649799
*Octopus bimaculoides* * Pickford & McConnaughey, 1949	MK649800–MK649803
*Octopus micropyrsus* * Berry, 1953	MK649804, MK649805
*Octopus veligero* * Berry, 1953	MK649806–MK649809
*Octopus chierchiae* * Jatta, 1889	MK649810, MK649811

**Note:**

* Sequences deposited for the purposes of this research.

**Figure 1:**
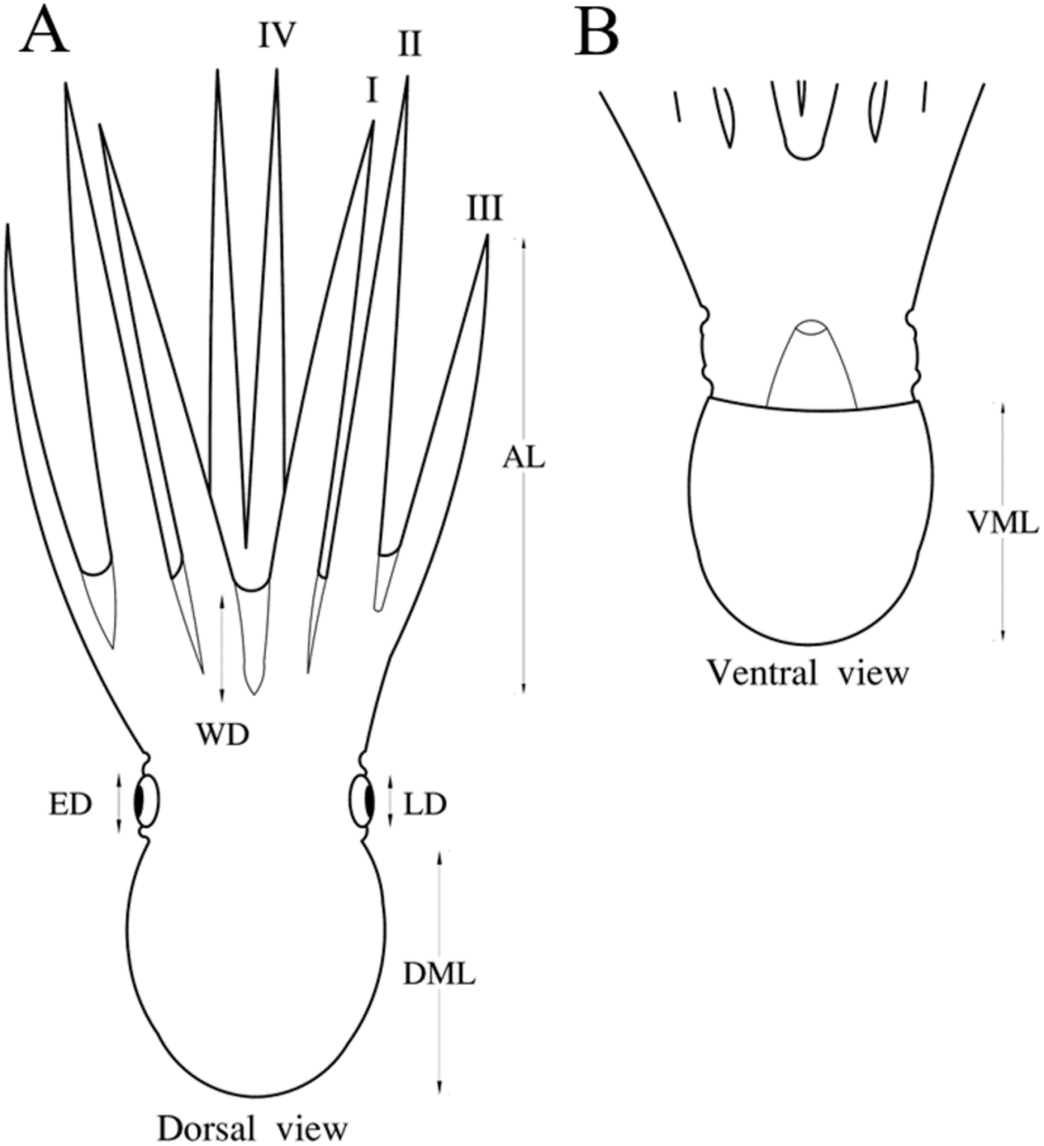
Nine body measurements of octopus suggested by Roper & Voss (1983). DML, dorsal mantle length; VML, ventral mantle length; WD, web depth; ED, eye diameter; LD, lens diameter (LD); and AIL, AIIL, AIIIL, AIVL, arm I–IV length. (A) Measurements of dorsal view. (B) Measurements of ventral view. Mariana Díaz-Santana-Iturrios drawed this figure.

A traditional morphometry analysis of body measurements was conducted considering all individuals, except for four octopuses that belonged to two species (*Octopus chierchiae* and *O. micropyrsus*). Nine measurements suggested by Roper & Voss (1983) were employed: dorsal mantle length (DML), ventral mantle length (VML), web depth, eye diameter (ED), lens diameter and arm I–IV length (AIL, AIIL, AIIIL, AIVL) (Fig. 1). Measurements were standardized according to DML in each group separately to remove the effect of size, using the allometric equation employed by Elliot, Haskard & Koslow (1995), included in Past v.2.12 software (Hammer, Harper & Ryan, 2001). A canonical variate analysis (CVA) was performed with the standardized matrix to quantify differences among groups. Statistical significance of the differences among groups was determined by Wilks’ lambda (λ). Grouping relationships among species based on the morphological similarity of body measurements were represented by a dendrogram that was built from Mahalanobis distances using the ascending hierarchical classification algorithm (Gower, 1967). These analyses were performed in XLSTAT software (Addinosoft, 2018).

## Results

**Figure 2:**
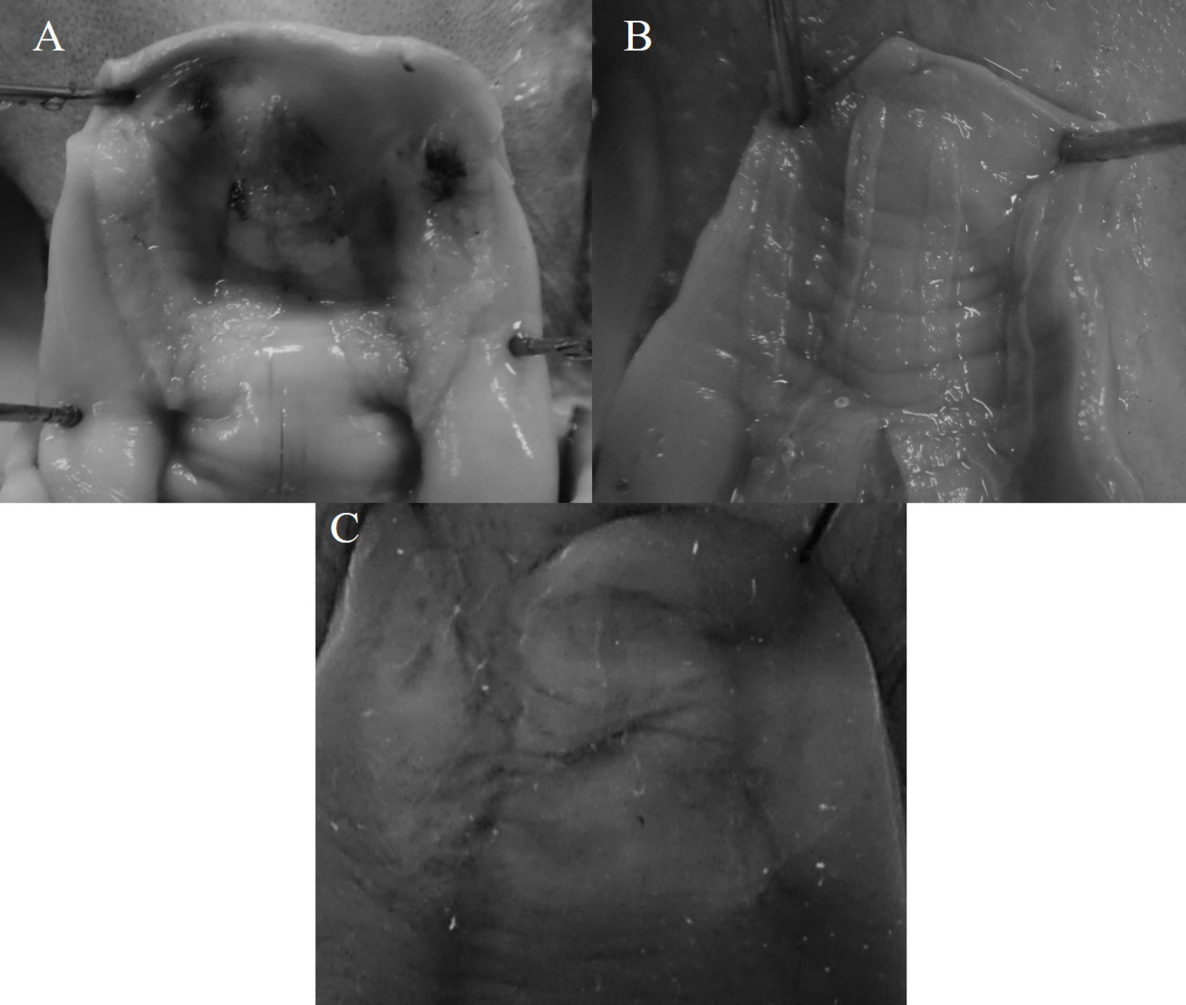
Funnel organ shapes found in species of the genus *Octopus* from the northeastern Pacific. (A) W. (B) V V. (C) IɅI.

In this study we evaluated the species-level assignment of octopuses from the northeastern Pacific using morphological, molecular, and morphometric criteria. According to the morphological characteristics, 8 of 11 species of the genus *Octopus* reported in the northeastern Pacific were identified from 270 individuals. The following funnel organ shapes (3) were detected among these taxa: W, V V and IɅI (Fig. 2).

*Octopus bimaculatus* Verrill, 1883

Material examined: 14 females (10.5–15.5 cm DML) and 16 males (9.5–13 cm DML) collected from artisanal fishery in Scorpion Bay, B.C.S. during February, 2016.

Diagnosis: arms moderately long. Arm formula 3 > 2 > 4 > 1. Web of moderate depth, deepest on lateral arms. Thin web margins extend to arm tips. Two to three hundred suckers on each arm. Some organisms with one or two enlarged suckers (from sucker 10 to 13). Funnel organ W-shaped. Eight to 10 lamellae per demibranch. Some specimens with supra-ocular papillae. Identification of this species was confusing due to its shared morphological similarity with *O. bimaculoides*. However, different from this latter species, in *O. bimaculatus*, the blue ring in each ocellus was comprised by broken chain links with distinct spokes radiating towards the outer dark spots surrounding each blue ring. In addition, identification was supported with the number of suckers on each arm and the sampling site (San Juanico, B.C.S.), where these species are not sympatric.

*Octopus chierchiae* Jatta, 1889

Material examined: one female (6.8 cm DML) collected from artisanal fishery in the Gulf of California at the coast of B.C.S. during 2006; one male (8.3 cm DML) sampled in an oceanographic cruise in Bahia Magdalena, B.C.S. during 2006.

Diagnosis: body rounded and smooth with continuous stripes that vary in size and shape along mantle, head, and arms. Long and narrow funnel. Web margins extend to near half the arm length. Arms long. Arm formula 4 > 3 > 2 > 1. Arm IV with around 40–41 suckers and arm I with 32. Six lamellae per demibranch. Funnel organ W-shaped.

*Octopus californicus* Berry, 1911

Material examined: 15 females (4.9–13.6 cm DML) and 21 males (2.5–12.6 cm DML) sampled in oceanographic cruises during 2004, 2005 and 2011 in Bahia Magdalena, B.C.S.

Diagnosis: although some individuals were in poor condition due to sampling, all diagnostic features were maintained and measurements were recorded. Specimens with large eyes and rough body texture. The skin is densely covered by minute star-like patches. Arm formula 2 > 3 > 1 > 4. Web deepest on lateral arms, margins extend to arm tips in ventral arms. Twelve to 13 lamellae per demibranch. Funnel V V-shaped. Males present a prominent ligula.

*Octopus bimaculoides* Pickford & McConnaughey, 1949

Material examined: 25 females (7.5–13 cm DML) and 25 males (11–14 cm DML) collected from artisanal fishery in Guerrero Negro, B.C.S. during September, 2006.

Diagnosis: individuals were highly similar to *O. bimaculatus*; thus, they were identified according to the number of suckers in each arm (140–190) and the shape of the blue rings in each ocellus, which presented well-defined chain links. Arm formula 2 > 3 > 4 > 1. Eight to 10 lamellae per demibranch. Funnel W-shaped.

*Octopus hubbsorum* Berry, 1953

Material examined: 21 females (6.1–18.5 cm DML) and 29 males (7.1–17.3 cm DML) collected from artisanal fisheries in Bahia de Matanchen, Nayarit during February, 2014; San Bruno, B.C.S. in July, 2014; Mazatlan, Sinaloa in January, 2015; Bahia Magdalena, B.C.S. in February, 2015; Acapulco, Guerrero in February, 2015; Guaymas, Sonora in May, 2015; San Juanico, B.C.S. in February, 2016; and Melaque, Jalisco and Santa Rosalia, B.C.S. in 2016.

Diagnosis: muscular species. Arm formula 3 > 4 > 2 > 1. Web of moderate depth, deepest in lateral arms. Each arm with 240 suckers. Most individuals presented 4–6 enlarged suckers in arms II and III. Nine to 11 lamellae per demibranch. Funnel organ W-shaped. Some individuals with four papillae in diamond pattern in the mid portion of dorsal mantle.

*Octopus alecto* Berry, 1953

Material examined: 36 females (2–7.5 cm DML) and 14 males (4.5–6.2 cm DML) collected in an oceanographic cruise in the coast of Guaymas, Sonora in August, 2015.

**Figure 3:**
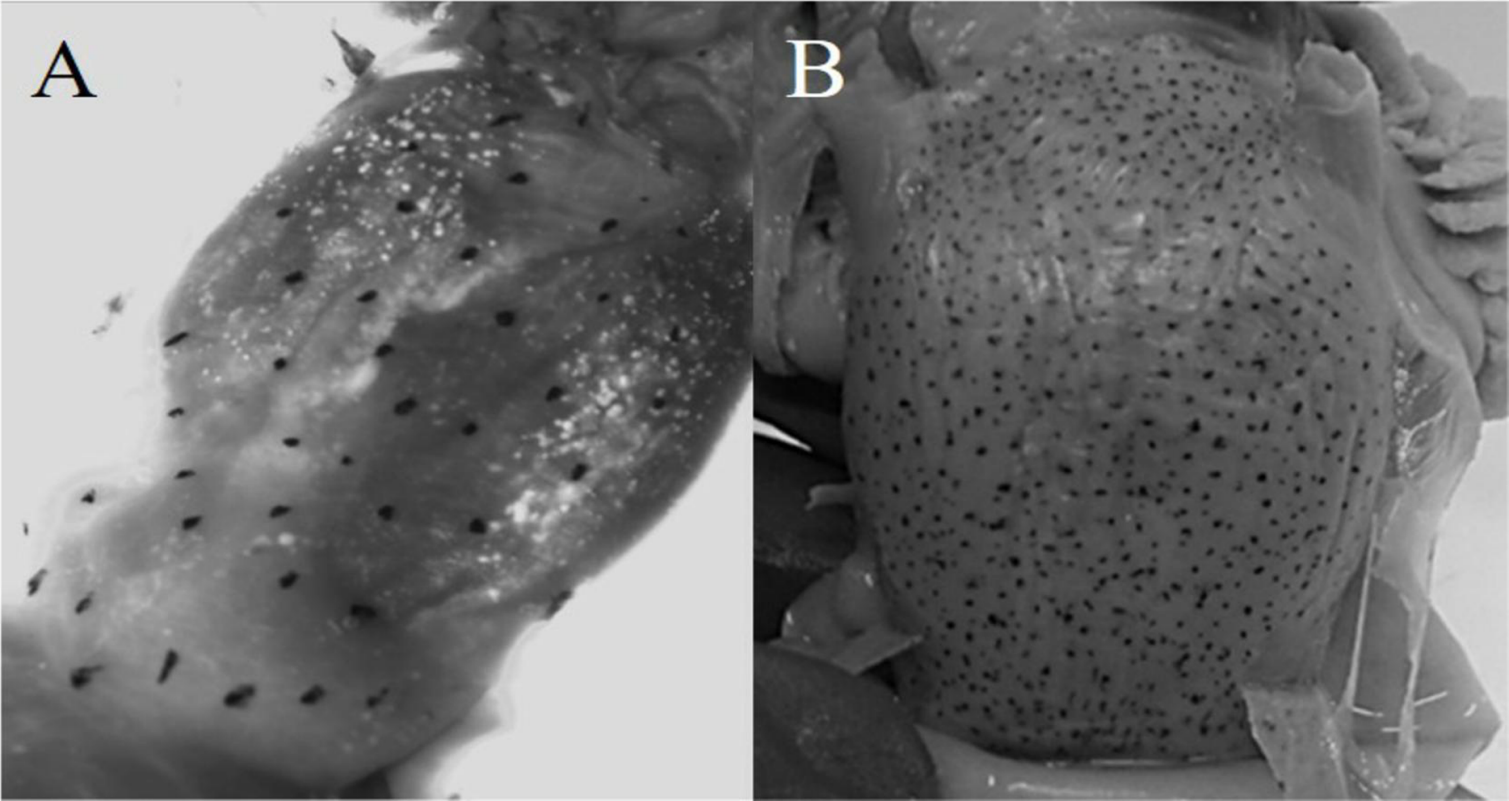
Chromatophores in the visceral sac. (A) *O. alecto*; (B) *O. veligero*.

Diagnosis: arm autotomy, lateral arms the longest. One hundred thirty to 133 suckers in each arm. Six to seven lamellae per demibranch. Funnel organ W-shaped. Whole body reddish, even in unfrozen individuals. Eyes densely covered by minute suckers. Visceral sac with chromatophores (Fig. 3).

*Octopus veligero* Berry, 1953

Material examined: 24 females (3.2–12.7 cm DML) and 26 males (2.7–10.3 cm DML) collected in oceanographic cruises during 2004, 2005, 2007, 2011 and 2012 in Bahia Magdalena, B.C.S.

Diagnosis: arms short, lateral arms the longest. Web thin and delicate, deepest in lateral arms. Arms with 120–160 suckers. Fifteen to 17 lamellae per demibranch. Funnel organ IɅI-shaped. Four dark spots in dorsal mantle. Visceral sac with chromatophores (Fig. 3).

*Octopus micropyrsus* Berry, 1953

Material examined: one female (6.3 cm DML) and one male (6.7 cm DML) collected from artisanal fishery during 2012 in Guerrero Negro, B.C.S.

Diagnosis: small and ovate mantle. Eyes large and prominent. Funnel organ W-shaped. Forty suckers in arms. Conical funnel with small opening. Six lamellae per demibranch.

**Figure 4:**
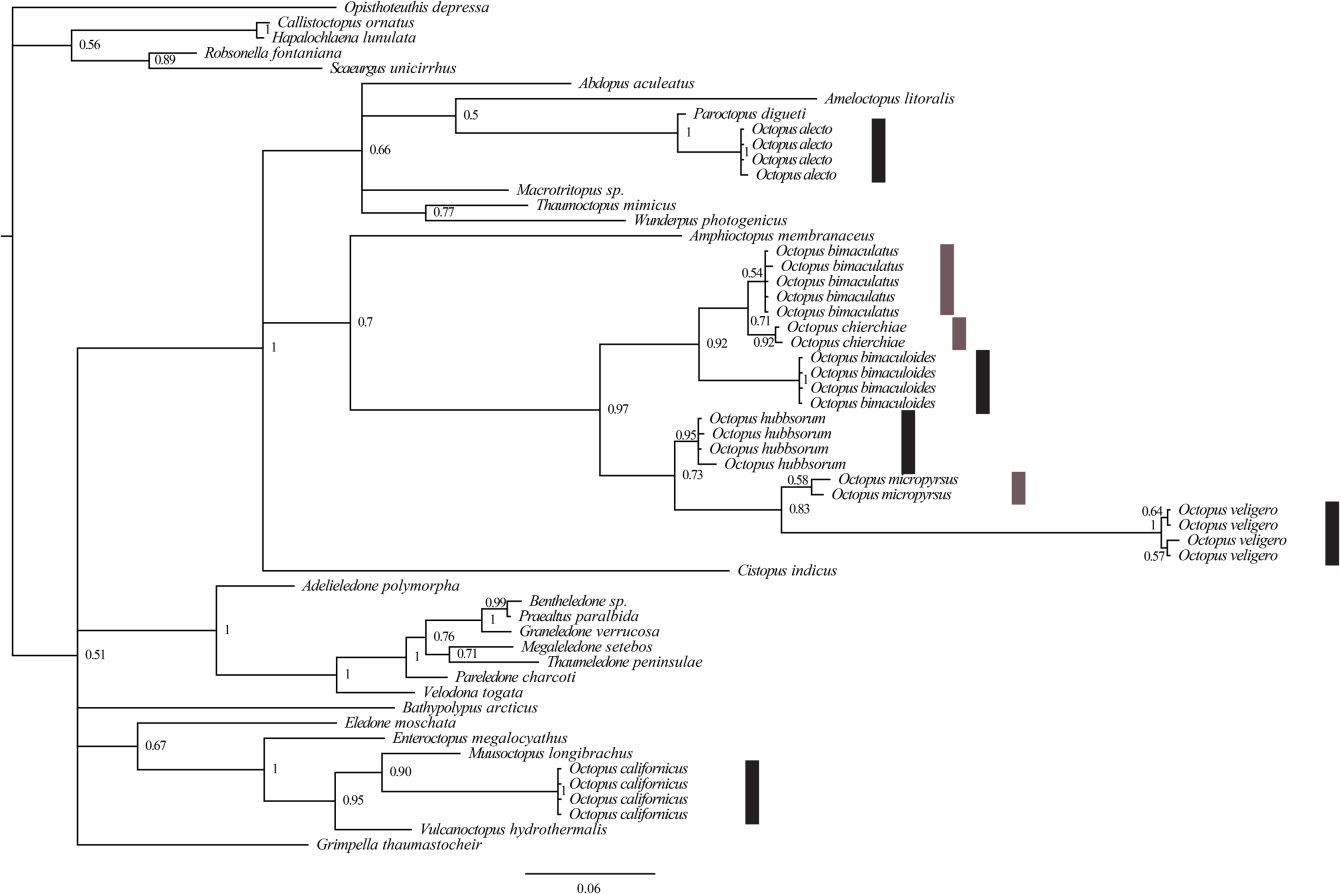
Molecular phylogeny of COI-gene sequences (420 bp) of species of the genus *Octopus* from the northeastern Pacific. Bars indicate a posteriori support values obtained from bPTP (dark gray > 0.85 and light gray < 0.85).

The COI-gene sequence analysis (420 bp: 235 conserved; 185 variable) revealed that the specimens evaluated (29) belonged to eight clades, these were associated to each of the eight species identified morphologically (Fig. 4). The bPTP also supported that these eight species identified a priori according to morphological characters were well-delimited molecularly, with no overlap among species, the lowest Bayesian support value was 0.671 for *O. bimaculatus* (Table 3). In addition, the molecular phylogeny of the COI-gene sequences analyzed (Fig. 4), indicated that the species evaluated in this study were catalogued into three genera: *Octopus alecto* in the genus *Paroctopus* Naef, 1923, *Octopus californicus* in the genus *Benthoctopus* Robson 1932, and the rest of the species in the genus *Octopus*.

**Table 3:** Species delimitation results according to the highest Bayesian supported solution of bPTP for octopuses of the genus *Octopus* from the northeastern Pacific.

Species	Bayesian support
*Octopus bimaculatus*	0.671
*Octopus chierchiae*	0.729
*Octopus californicus*	0.925
*Octopus bimaculoides*	0.912
*Octopus hubbsorum*	0.916
*Octopus alecto*	0.880
*Octopus veligero*	0.984
*Octopus micropyrsus*	0.719

**Figure 5:**
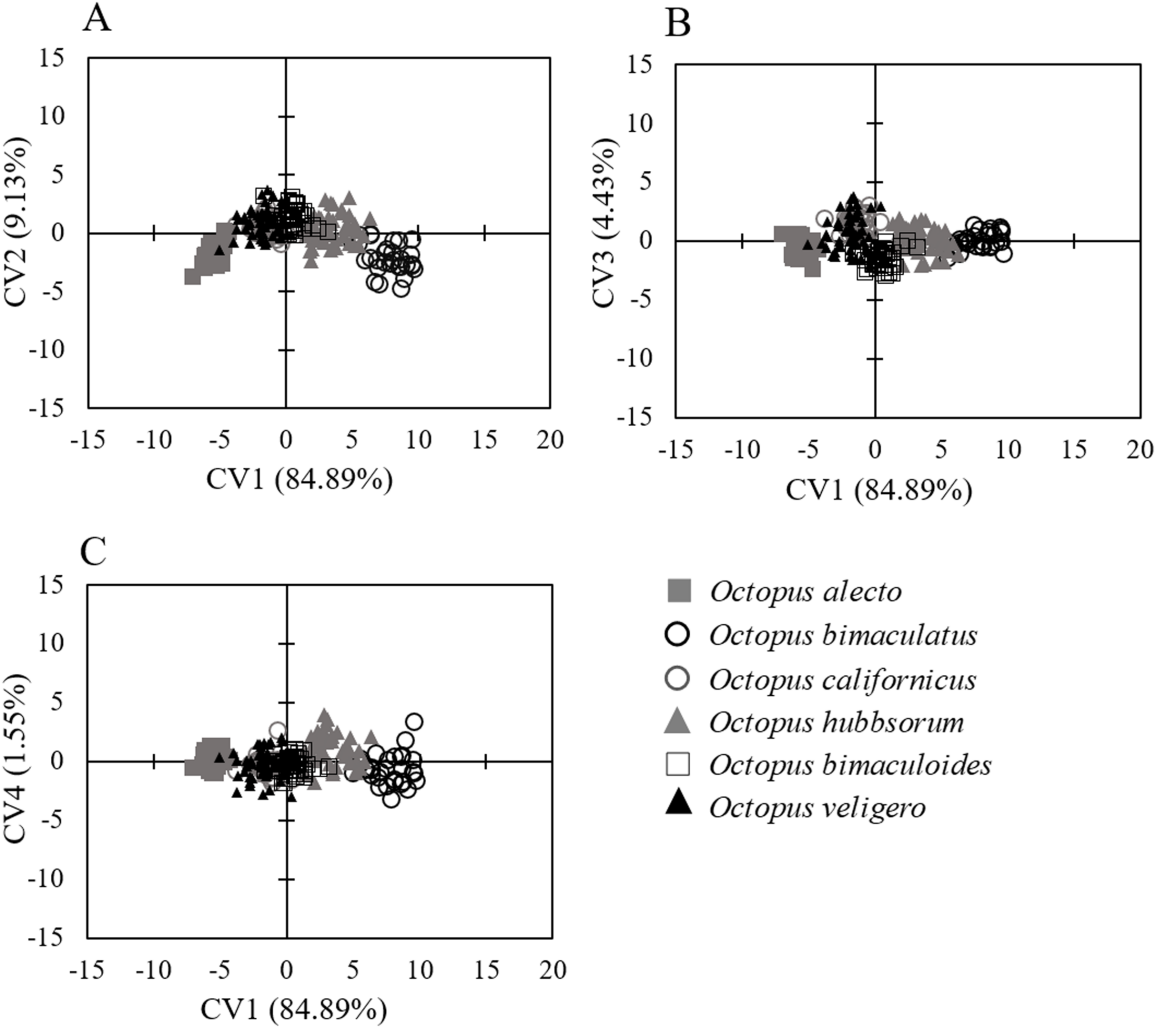
Canonical variate analysis of body measurements of species of the genus Octopus from the northeastern Pacific standardized according to DML. (A) CV1 and CV2. (B) CV1 and CV3. (C) CV1 and CV4.

**Figure 6:**
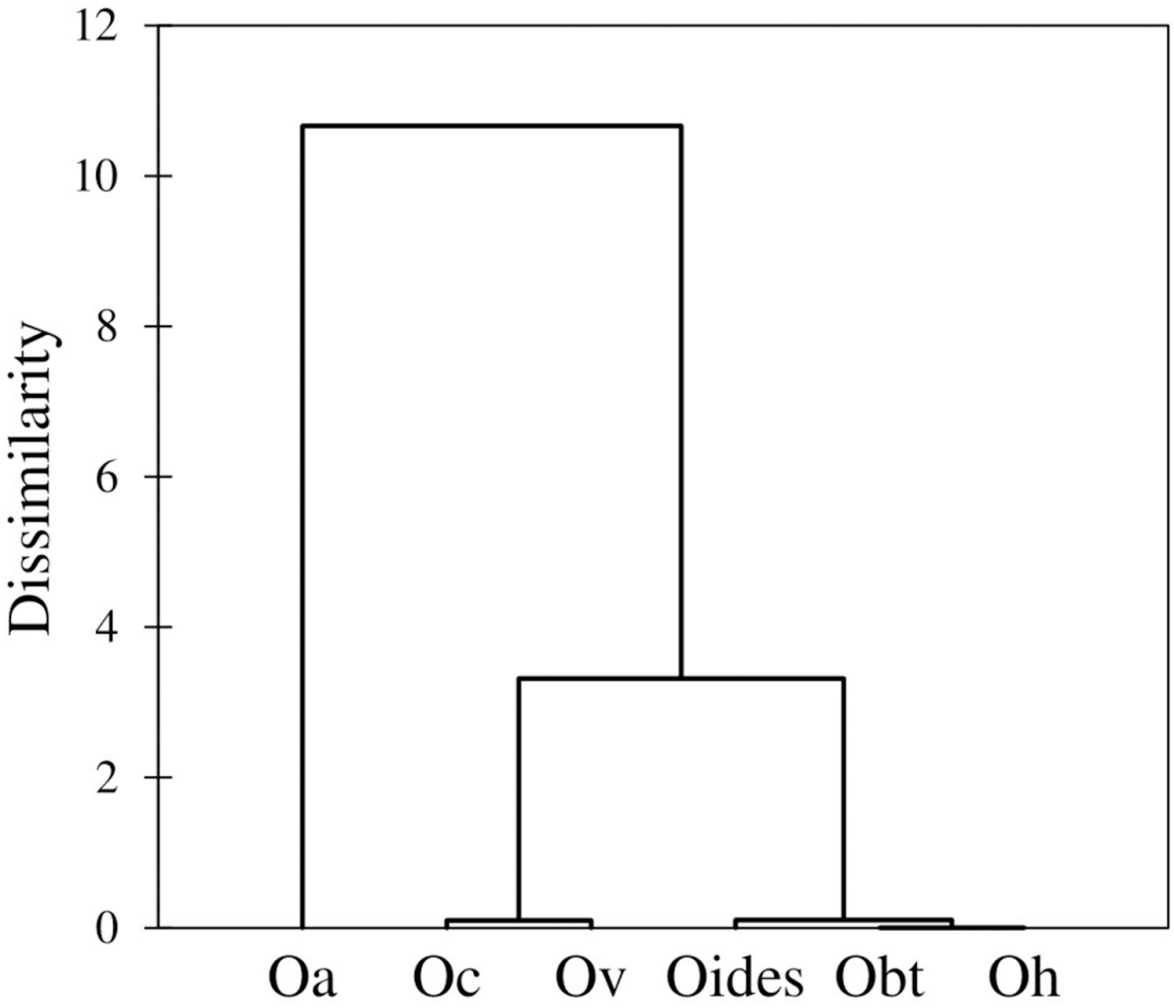
Dendrogram based on the Mahalanobis distances obtained from the traditional morphometry analysis of body measurements of octopuses of the genus *Octopus* from the northeastern Pacific.

Similarly, the analysis of body measurements revealed the existence of six taxa, these corresponded to the species identified according to morphological attributes and the COI-sequence analysis; the remaining species (*Octopus chierchiae* and *O. micropyrsus*) were not included in the morphometric analysis due to the low number of individuals representing each of the two taxa (*n* = 2, respectively), as stated earlier. Four canonical variables explained the total variance among groups: CV1 = 84.89%; CV2 = 9.13%; CV3 = 4.43%; CV4 = 1.55% (Fig. 5). Significant differences were found among the species identified (λ = 0.009; *p* = 0.0001). Despite the correct assignment matrix indicated overlap among groups, the lowest assignment was 80% for *O. veligero*, which strongly supports the divergence among groups (Table 4). Arm I–IV lengths were the variables that showed the highest loading for CV1 (Table 5), *O. bimaculatus* presented the largest arms according to DML (4.94 times DML in Arm III), while the smallest arm proportions were detected in *O. veligero* (2.17 times DML in Arm I to 2.34 times DML in arm II) (Table 6). The pattern of morphological similarity within each group obtained from Mahalanobis distances indicated that *O. alecto* was the most divergent species and *O. bimaculoides, O. bimaculatus* and *O. hubbsorum* were the most morphometrically similar taxa (Fig. 6).

**Table 4:** Classification matrix of octopuses of the genus *Octopus* from the northeastern Pacific on the basis of body measurements.

Number of classified octopuses								
Species	Oa	Obt	Oc	Oh	Oides	Ov	Total	%Correct
Oa	50	0	0	0	0	0	50	100
Obt	0	29	0	1	0	0	30	96.67
Oc	0	0	35	0	1	0	36	97.22
Oh	0	0	0	48	2	0	50	96
Oides	0	0	0	1	49	0	50	98
Ov	1	0	6	0	3	40	50	80

**Note:**

Oa, *Octopus alecto*; Obt, *O. bimaculatus*; Oc, *O*. californicus; Oh, *O. hubbsorum*; Oides, [i]O. bimaculoide.

**Table 5:** Correlation matrix of variables and factors of CVA of octopuses of the genus *Octopus* from the northeastern Pacific.

Measurement	CV1	CV2	CV3	CV4
Ventral mantle length	0.940	**0.320**	−0.052	**0.077**
Web depth	0.913	−0.043	**0.118**	**−0.261**
Eye diameter	0.742	0.106	**0.577**	**0.247**
Lens diameter	0.324	**0.347**	**0.732**	**−0.330**
Arm I length	**0.959**	−0.133	**0.081**	−0.027
Arm II length	**0.959**	−0.144	−0.009	−0.046
Arm III length	**0.963**	**−0.208**	−0.013	−0.057
Arm IV length	**0.963**	**−0.190**	0.006	0.068

**Note:**

Variables with highest loading are highlighted in bold.

**Table 6:** Proportion of arm I–IV lengths according to DML (in number of times) of octopuses of the genus *Octopus* from the northeastern Pacific.

Species	AIL/DML	AIIL/DML	AIIIL/DML	AIVL/DML
Oa	2.39	2.51	**2.67**	2.55
Obt	3.97	4.67	**4.94**	4.51
Oc	2.93	**3.15**	2.95	2.73
Oh	2.79	3.02	**3.24**	3.14
Oides	2.27	**2.67**	2.66	2.44
Ov	2.17	**2.34**	2.33	2.27

**Note:**

The highest proportion for each species is highlighted in bold. Oa, *Octopus alecto*; Obt, [i]O. bim.

## Discussion

In this study, we evaluated the identity of octopuses of the genus *Octopus* from the northeastern Pacific using different taxonomic tools. Three of the 11 species reported for the area (*O. fitchi*, *O. penicillifer* and *O. rubescens*) were not assessed in our study. Their absence can be due to limitations related to the sampling sites, given that type localities were not considered (*O. fitchi* = Punta San Felipe, Baja California; *O. penicillifer* = Punta Arena, Baja California Sur; *O. rubescens* = Isla Coronado, Baja California) (Berry, 1953; Jereb et al., 2016). However, the diagnostic characteristics of these three species are well-delimited according to literature, except for the number of lamellae per demibranch, which is not determined in *O. fitchi* and *O. penicillifer* (Berry, 1953, 1954; Jorgensen, 2009; Sweeney, Roper & Hochberg, 1988; Roper & Mangold, 1991; Jereb et al., 2016).

The original descriptions of species of the genus *Octopus* were developed considering fresh individuals, and therefore, Hochberg (1998) suggested that identification of these taxa should be performed using individuals that were recently caught in order to avoid confusions related to morphological deformations that could derive from the preservation methods. In practice, however, this is difficult to accomplish, given that at least in Mexico (sampling area for this study), octopuses are captured almost exclusively from fisheries, and freezing them is essential to maintain the fishery products in optimal quality (CONAPESCA, 2008). The specimens that we evaluated were not fresh, the biological material deposited in the local collection CIBNOR-CEFACIB was fixated in formaldehyde 4% and preserved in ethanol 96% (*n* = 40), except for individuals of *O. veligero*, which were stored frozen (*n* = 50); and the rest of the specimens (*n* = 180), collected in artisanal fisheries, were stored frozen. All octopuses were identified to the species level according to the morphological criteria specified in the original descriptions, and there was no morphological variation that allowed to suspect the presence of non-described species. In contrast, in the western coast of the Baja California Peninsula, Hochberg (1976) detected two species that were not described. According to our observations, the species analyzed in this study are well-delimited in their diagnostic morphological characters. Thus, we consider that for these species, the root of the taxonomic problems is more related to the lack of informative attributes at the genus level, as discussed below.

In the family Octopodidae, the morphological characteristics are widely shared among genera, for instance, the most important attributes to catalog species into the genus *Octopus* are the presence of an ink sac and suckers in a two-row arrangement in each arm (Cuvier, 1797; Guzik, Norman & Crozier, 2005; Jereb et al., 2016); however, these characteristics are detected in 17 of the 39 genera of the family Octopodidae, and from these taxa, three species of three genera within the family can be found in the sampling area (Mexican Pacific) (Norman & Hochberg, 2005; Jereb et al., 2016). Another broadly accepted character for the genus *Octopus* is the presence of a pair of white spots in the dorsal mantle; this feature was detected by Packard & Sanders (1971) exclusively for *O. vulgaris*, and it was later used to identify *O. bimaculatus*, *O. hubbsorum*, *O. rubescens*, *O. bimaculoides*, and species of other genera within the Octopodidae (Packard & Hochberg, 1977; Jereb et al., 2016). Nevertheless, we did not detect this character in the specimens analyzed, this can be due to the fact that, different from our study, the octopuses evaluated by Packard & Sanders (1971) and Packard & Hochberg (1977) were alive and held in aquariums when the coloration patterns were described by these authors.

Moreover, the funnel organ shape is part of the morphological attributes standardized for inter-generic discrimination, this structure is difficult to detect (if not impossible) in defrosted or badly preserved material (Jereb et al., 2016). We were not able to observe the funnel-organ shape in some individuals evaluated in this research, although it did not limit the taxon determination. A single funnel organ shape (W) is considered in the diagnosis of the genus *Octopus* (Jereb et al., 2016). However, *O. californicus* and *O. veligero* present particular funnel-organ shapes (V V and IɅI, respectively), hence, these features must be included in the diagnosis of the genus *Octopus*, as is described for *Eledone* Leach, 1817, *Benthoctopus* Grimpe, 1921, *Pareledone* Robson, 1932 and *Callistoctopus* Taki, 1964, which include species with different funnel-organ shapes, or else, for *O. californicus*, this character should be employed to support its generic re-assignation, as is furtherly discussed.

Similarly, the presence of chromatophores in the visceral sac of *O. alecto* and *O. veligero* is only described in their diagnoses at paralarval stage (Sweeney et al., 1992), as occurs for *O. fitchi* and *O. rubescens* (the latter two species not evaluated in this study). Due to the taxonomic and systematic problems associated to the genus *Octopus* (Guzik, Norman & Crozier, 2005), the presence of chromatophores in the visceral sac at adult stage could be informative for inter-generic classification, especially since *O. alecto* and *O. veligero* (as well as *O. rubescens*) are provisionally assigned to the genus *Octopus* (Norman & Hochberg, 2005), and considering that our molecular and morphometric results indicated that *O. alecto* does not belong to the genus *Octopus*.

In this research, we employed a phylogenetic tree to represent the species identification according to partial COI-gene sequences. In this regard, and also according to the species delimitation obtained by bPTP, the octopuses analyzed belong to eight species. However, rescuing the phylogenetic trace of this analysis, in combination with our morphological and morphometric observations, it became evident that *O. alecto* and *O. californicus* belong to two genera different from *Octopus*. Strugnell et al. (2011) performed molecular analyses with different markers (four mitochondrial and one nuclear) and found that *O. californicus* was catalogued in a clade containing *Benthoctopus*, and with this data, the authors confirmed that the latter genus originated in the northern hemisphere, although no inter-generic re-assignation was suggested for *O. californicus* in their research. Therefore, this species remained in the category of provisionally assigned into the genus *Octopus* (Jereb et al., 2016). Concordantly, Ibáñez et al. (2018) also found that *O. californicus* belonged to a genus different from *Octopus* that is, catalogued in the family Enteroctopodidae Strugnell et al. (2014).

The dendrogram obtained in the morphometric assessment corresponds almost entirely to the phylogenetic clustering of the species under study, except for *O. californicus* and *O*. *veligero*, which bifurcate from the same branch. Voight (1991) noted that despite the soft nature of the octopus body plan, the preservation methods influence the intra-specific variation of shape only at a minimal scale, which is similar to our research, where the fixation/preservation methods did not limit the morphometric evaluation. Concordant to our study, Voight (1994) found correspondence between entities detected a priori and those grouped a posteriori, once the morphometric analysis was performed. This author included the following *Octopus* species from the northeastern Pacific in her research: *O. bimaculatus*, *O. chierchiae*, *O. alecto*, *O. fitchi*, *O. hubbsorum* and *O. penicillifer*, and related the morphometric differences among species with their types of habitat, whether associated to sandy bottoms or rocky reefs. Accordingly, in our study, three groups of species were detected in the dendrogram, the first was represented by *O. alecto*, which lives in shallow depths (0–4 m) in estuarine sediments; the second group was comprised by *O. californicus* and *O. veligero*, both taxa live in deeper waters (100–900 m and 90–200 m, respectively) in soft mud and muddy sand substrates; and the third was constituted by the species of commercial interest, *O. bimaculoides*, *O. bimaculatus* and *O. hubbsorum*, which live in 0–50 m depths and are typically associated to rocky reefs (Berry, 1953; Jereb et al., 2016). Thus, according to our results, the morphometric relationships among taxa are determined by their habitat, and considering the phylogenetic tree built with partial COI-gene sequences, these relationships derive from adaptations rather than ancestral heritage.

The Bayesian support separating *O. bimaculatus* from *O. chierchiae* would suggest that these two species are morphologically similar. In contrast, *O. chierchiae* presents a particular morphology among the species evaluated in our study (presence of continuous stripes along mantle, head, and arms and absence of ocelli), while *O. bimaculatus* is characterized by a pair of ocelli and no stripes; in fact, the high morphological similarity between this species and *O. bimaculoides* reaches a point where these two species are easily confused (Jereb et al., 2016). Debenedetti et al. (2014) detected that the genotypic variability is not correlated with the morphological differences among species of the genus *Octopus*, which is concordant with what we found in regard to these three species. In addition, *O. chierchiae* and *O bimaculoides* are holobenthic octopuses that produce large eggs and hatchlings with direct development (Rodaniche, 1984; Ibarra-García et al., 2018), different from *O. bimaculatus*, which is merobenthic and produces numerous small eggs that hatch into planktonic paralarvae (Jereb et al., 2016). Thus, our results contrast with Ibáñez et al. (2018) in two aspects, the first is that *O. chierchiae* is not found at high latitudes and does not live in cold environments (Jereb et al., 2016), as is expected for its type of development and egg number; and the second is that our tree did not cluster phylogenetic groups according to the type of development and egg size, although this result is most certainly related to the fact that we only employed one mitochondrial DNA marker and no environmental and/or qualitative variables to complement the phylogenetic tree due to the objective of our study, which was to assess the biodiversity of *Octopus* from the northeastern Pacific. In this regard, although the use of additional molecular techniques (genome-wide approaches) were recommended by Amor et al. (2019), as they were able to detect cryptic species within the *O. vulgaris* complex, in our study we focused on incorporating information from different sources, which allowed us to corroborate the existence of eight taxa, however, we agree that more profound molecular analyses should be addressed in further studies to fully understand the evolutionary processes of the octopuses under study.

In addition, Voight (1994) found that the lengths of arms I–IV are relevant to discriminate most species, although she did not find a clear relationship between these measurements and the DML in *O. bimaculoides*. Considering the results of our study, other measurements such as VML and ED (greatest loading in CV2), are more informative for this species. Moreover, the genus *Octopus* is characterized by arm lengths that are typically 3–5 times the DML (Jereb et al., 2016), however, according to our findings, only *O. bimaculatus* is in full compliance with this statement. The arm lengths of *O. alecto*, *O. veligero* and *O. bimaculoides* range 2–3 times DML and this length proportion is a typical character of the genera *Amphioctopus* Fischer, 1882 and *Paroctopus*, which are also represented by species with an ink sac, suckers in a two-row arrangement, and a W-shaped funnel organ (Jereb et al., 2016). In consequence, the proportion of arm length in regard to DML is relevant to discriminate the species evaluated in this research, and although this feature resulted ambiguous for *Octopus* according to our analysis, it supports that *O. alecto* belongs to the genus *Paroctopus* and that *O. californicus* (2–3.5 times DML) belongs to a genus different from *Octopus* within the family Enteroctopodidae, although further studies should be performed to formalize their respective novel combinations.

Furthermore, Amor et al. (2017) performed a molecular analysis of COI gene sequences and a morphometric evaluation to assess the *Octopus vulgaris* species complex, and similar to our study, these authors found correspondence in the determination of taxa between both analyses, although they included measurements of the hectocotylized arm and its parts, and found that sucker counts of this modified arm explained most of the variation among groups. In our research, we decided not to employ sexual characters in the morphometric analysis given that sex-specific features would only provide partial information regarding the species. In addition, variations in hectocotyli were recently detected in *O. hubbsorum* by Díaz-Santana-Iturrios et al. (2019), which compromises the validity of this structure as diagnostic character for the species under study.

The octopuses evaluated in this research were collected in the Mexican Pacific, the bathymetry of this area is widely variable, 80% of the seafloor exceeds 2,000 m depths and 6% is at depths shallower than 200 m (Espinosa, 2004). The Mexican Pacific is influenced by the cold California Current in the western coast of the Baja California Peninsula (Ortiz & De La Lanza, 2006). The Gulf of California has contact with the adjacent Pacific Ocean (Álvarez-Borrego & Galindo-Bect, 1975), and freshwater inputs flow into its southern portion and the rest of the Mexican Pacific (De La Lanza, Ortiz & Carbajal, 2013). The high diversity of octopuses from the northeastern Pacific confirmed in this study suggests that despite the varied depths and influence of diverse water masses in the sampling area, the environmental conditions are not hostile compared to low latitudes, where biodiversity drops off sharply (Collins et al., 2019). Therefore, our study constitutes an important reference to monitor octopod biodiversity in light of the incipient climate change.

## Conclusions

According to the features and taxa considered in our taxonomic evaluation, the species of the genus *Octopus* from the northeastern Pacific are morphologically, molecularly and morphometrically well-delimited. The funnel organ shapes of the species assessed should be included in the diagnosis of *Octopus* or else should be used to support the re-assignation of species at the genus level. According to the phylogenetic relationships obtained from the analysis of partial COI-gene sequences, *O. alecto* belongs to the genus *Paroctopus* and *O. californicus* to a genus in the family Enteroctopodidae, although these generic re-assignations should be formalized in further studies. The morphometry analysis indicated that morphometric relationships are determined by the type of habitat; in addition, the ancestral trace of these features should be assessed in further studies. Arm I–IV lengths are relevant attributes to discriminate the species under study and are informative for the generic re-assignation of *O. alecto* and *O. californicus*.

## Supplemental Information

10.7717/peerj.8118/supp-1 Supplemental Information 1 Partial COI gene sequences employed in the molecular analysis.

10.7717/peerj.8118/supp-2 Supplemental Information 2 Body measurements of octopuses employed in the traditional morphometry analysis.

## Additional Information and Declarations

### Data Availability

The following information was supplied regarding data availability: The body measurements of octopuses employed in the traditional morphometry analysis and the partial COI gene sequences are available in Supplemental Files and GenBank: MK649783 to MK649811.
